# Association of Posttraumatic Stress and Depressive Symptoms With Mortality in Women

**DOI:** 10.1001/jamanetworkopen.2020.27935

**Published:** 2020-12-04

**Authors:** Andrea L. Roberts, Laura D. Kubzansky, Lori B. Chibnik, Eric B. Rimm, Karestan C. Koenen

**Affiliations:** 1 Harvard T.H. Chan School of Public Health, Boston, Massachusetts

## Abstract

**Importance:**

Consistent evidence has found associations between posttraumatic stress disorder (PTSD) and increased risk of chronic disease and greater prevalence of health risk factors. However, the association between PTSD and all-cause mortality has not been thoroughly investigated in civilians.

**Objective:**

To investigate the association between PTSD symptoms, with or without comorbid depressive symptoms, and risk of death.

**Design, Setting, and Participants:**

This prospective cohort study was conducted using data on female US nurses in the Nurses’ Health Study II followed up from 2008 to 2017. Women who responded to a 2008 questionnaire querying PTSD and depressive symptoms were included. Data were analyzed from September 2018 to November 2020.

**Exposures:**

Symptoms of PTSD, measured using the short screening scale for *Diagnostic and Statistical Manual of Mental Disorders* (Fourth Edition) PTSD, and depression symptoms, measured using the Center for Epidemiologic Studies Depression Scale–10 in 2008.

**Main Outcomes and Measures:**

All-cause mortality was determined via National Death Index, US Postal Service, or report of participant’s family. The hypothesis being tested was formulated after data collection. Trauma exposure and PTSD symptoms were jointly coded as no trauma exposure (reference), trauma and no PTSD symptoms, 1 to 3 PTSD symptoms (subclinical), 4 to 5 PTSD symptoms (moderate), and 6 to 7 PTSD symptoms (high).

**Results:**

Among 51 602 women (50 137 [97.2%] White individuals), the mean (range) age was 53.3 (43-64) years at study baseline in 2008. PTSD and probable depression were comorbid; of 4019 women with high PTSD symptoms, 2093 women (52.1%) had probable depression, while of 10 105 women with no trauma exposure, 1215 women (12.0%) had probable depression. Women with high PTSD symptoms and probable depression were at nearly 4-fold greater risk of death compared with women with no trauma exposure and no depression (hazard ratio [HR], 3.80; 95% CI, 2.65-5.45; *P* < .001). After adjustment for health factors, women with these conditions had a more than 3-fold increased risk (HR, 3.11; 95% CI, 2.16-4.47, *P* < .001). Women with subclinical PTSD symptoms without probable depression had increased risk of death compared with women with no trauma exposure and no depression (HR, 1.43; 95% CI, 1.06-1.93; *P* = .02). Among 7565 women with PTSD symptoms and probable depression, 109 deaths (1.4%) occurred for which we obtained cause of death information, compared with 124 such deaths (0.6% ) among 22 215 women with no depression or PTSD symptoms. Women with PTSD symptoms and probable depression, compared with women with no PTSD or depression, had higher rates of death from cardiovascular disease (17 women [0.22%] vs 11 women [0.05%]; *P* < .001), diabetes (4 women [0.05%] vs 0 women; *P* < .001), unintentional injury (7 women [0.09%] vs 7 women [0.03%]; *P* = .03), suicide (9 women [0.12%] vs 1 woman [<0.01%]; *P* < .001), and other causes of death (14 women [0.19%] vs 17 women [0.08%]; *P* = .01).

**Conclusions and Relevance:**

These findings suggest that at midlife, women with high PTSD symptoms and co-occurring probable depression are at increased risk of death compared with women without these disorders. Treatment of PTSD and depression in women with symptoms of both disorders and efforts to improve their health behaviors may reduce their increased risk of mortality.

## Key Points

**Question:**

Are comorbid posttraumatic stress disorder (PTSD) and depressive symptoms associated with mortality risk in women?

**Findings:**

In this cohort study of 51 602 women followed up for 9 years, those with high PTSD symptoms and comorbid-probable depression had 3.8-fold increased risk of death compared with women without trauma exposure or probable depression. Decreased body mass index, nonsmoking status, physical activity, and being married were associated with decreased risk of death.

**Meaning:**

These findings suggest that treatment of PTSD and depression in women with symptoms of both disorders and efforts that improve their health behaviors may reduce the increased risk of mortality among this population.

## Teaser

This cohort study examines the association between posttraumatic stress disorder, with and without comorbid depression, and mortality in women.

## Introduction

Posttraumatic stress disorder (PTSD) has been associated with increased risk of chronic disease, including hypertension, cardiovascular disease, and type 2 diabetes,^1,2,3,4,5^ and with greater prevalence of health risk factors, such as obesity and smoking.^6,7^ Furthermore, PTSD has been associated with biological changes involved in several disease processes,^8^ including hypothalamic-pituitary-adrenal–axis alterations and related inflammation and immune dysregulation,^9,10,11^ oxidative stress,^12^ poor sleep,^13^ and indicators of accelerated aging.^5,14^ These diseases, health risk factors, and biological changes are associated with increased mortality.^15^ However, the association between PTSD and all-cause mortality has been investigated almost exclusively in samples of male military veterans. In these studies,^16,17,18,19,20,21,22^ PTSD has been associated with increased risk of all-cause mortality as well as mortality from cardiovascular disease, cancer, and external causes, such as unintentional and intentional injury, with few exceptions.^23^ However, PTSD occurs at higher rates among women. In the United States, lifetime prevalence of PTSD in women is more than 2-fold that of men (9.7% vs 3.6%).^24^ Risk of PTSD following a trauma exposure is similarly 2-fold higher in women compared with men.^25,26^ Only 1 large study,^27^ using Danish medical records, has investigated the association of PTSD with mortality in women or civilians, finding more than 2-fold the risk of death in individuals with PTSD vs those without PTSD.

Depression often co-occurs with PTSD^28^ and, like PTSD, it is far more prevalent in women than men.^29,30^ Depression has been independently associated with greater prevalence of health risk factors^31,32^ and risk of mortality.^33,34,35,36^ Evidence also suggests that PTSD with depression may constitute a particularly severe subtype of posttraumatic response, with unique biological outcomes important for physical health.^37^ Thus, PTSD with depression may be associated with even greater risk of mortality compared with PTSD alone. However, to our knowledge, only 3 relatively short-term studies have examined this possibility. One study,^38^ among Japanese earthquake survivors ages 65 years and older, found increased risk of all-cause mortality over 3.3 years of follow-up among individuals who had depression at baseline, regardless of whether they had high PTSD symptoms, compared with individuals who did not have depression at baseline. Individuals with PTSD and depression were not at significantly increased risk of dying during the follow-up period compared with those with depression only. A 2013 study^39^ of 391 patients with end-stage kidney disease found increased risk of death among patients with both depression and PTSD and patients with depression alone but not among those with PTSD alone, compared with patients with neither disorder, in 3.5 years of follow-up. A 2010 study^40^ of in-hospital mortality after a coronary artery bypass grafting surgical treatment found that patients with comorbid depression and PTSD were at increased risk of death compared with patients with neither disorder. Patients also had an increased risk of death if they had depression alone or PTSD alone.

Several studies^6,31,32,41^ have found that individuals with PTSD or depression, compared with individuals without these disorders, have increased prevalence of health risk factors, such as smoking and obesity, which may contribute to increased mortality. Thus, higher prevalence of health risk factors in individuals with co-occurring PTSD and depression may account for possible increased mortality. It remains largely unknown whether PTSD is associated with increased mortality among civilians and women, whether co-occurring depression is associated with further increased risk, and whether health-risk factors are associated with these increased risks of death. In the present study, we examined the association of PTSD and depression symptoms with risk of death in a large prospective cohort of women, the Nurses’ Health Study II.^42^ We further examined whether health-related factors, including body mass index (BMI; calculated as weight in kilograms divided by height in meters squared), smoking, and exercise, were associated with differences in mortality among individuals with PTSD or depression.

## Methods

This cohort study examined women in the Nurses’ Health Study II. The institutional review board of Brigham and Women’s Hospital approved that study’s protocol. Return of the questionnaire by the respondent via US mail constituted implied informed consent. The Nurses’ Health Study II is an ongoing cohort study of 116 429 women, enrolled in 1989 at ages 25 to 42 years (median age, 34.0 years) and followed biennially. In 2008, 60 804 women who completed the most recent biennial questionnaire and an earlier supplemental questionnaire were mailed a supplemental PTSD questionnaire, with 54 687 women responding. As the Nurses’ Health Study II was initially formed to study the health effects of oral contraceptive use, only women were enrolled.

### Measures

#### Trauma, PTSD, and Depression

For each of 15 potentially traumatic events (eg, serious motor vehicle crash) and an additional other event, women in the study reported in 2008 whether they had ever experienced the event. They were asked which event they considered their worst or most distressing event. Seven PTSD symptoms in relation to this worst event were queried with the Short Screening Scale for *Diagnostic and Statistical Manual of Mental Disorders* (Fourth Edition)^43^ PTSD.^44^ Trauma exposure and PTSD symptoms were jointly coded as no trauma exposure (reference), trauma and no PTSD symptoms, 1 to 3 PTSD symptoms (subclinical), 4 to 5 PTSD symptoms (moderate), and 6 to 7 PTSD symptoms (high). In a representative sample of Detroit residents ages 18 to 45 years,^44^ a cutoff of 4 or more identified PTSD cases with sensitivity of 80%, specificity of 97%, positive predictive value of 71%, and negative predictive value of 98%, and a cutoff of 6 or more identified cases with sensitivity of 38%, specificity of 99%, positive predictive value of 87%, and negative predictive value of 95%. We additionally coded PTSD symptoms as a continuous variable (range, 0-7). Past-week depressive symptoms were assessed in 2008 using the Center for Epidemiologic Studies Depression Scale–10 (CESD-10)^45^ and dichotomized at 10 or more to indicate probable depression.^45^ We additionally coded depressive symptoms as a continuous variable (range, 0-30). The CESD-10 has been validated against the highly validated longer form, the Center for Epidemiologic Studies Depression Scale–20, in a sample of older adults in a US health-maintenance organization (Cohen κ, 0.97)^45^ and against clinical evaluations with good psychometrics.^46,47^

To examine the co-occurrence of PTSD and depression with mortality, we also characterized PTSD and depression with an interaction term, using indicator variables as follows: no depression or trauma (reference), trauma with no depression and no PTSD symptoms, no depression and 1 to 3 PTSD symptoms, no depression and 4 to 5 PTSD symptoms, no depression and 6 to 7 PTSD symptoms, depression and trauma with no PTSD symptoms, depression and 1 to 3 PTSD symptoms, depression and 4 to 5 PTSD symptoms, and depression and 6 to 7 PTSD symptoms.

#### Mortality

Mortality through December 2017 and cause of death were ascertained from family members, the National Death Index, cancer registries, and the US Postal Service. Owing to time lags between report of death and ascertainment and coding of death record data, cause of death was available for 384 of 555 women (69.1%) who died during follow-up.

#### Health-Related Factors and Covariates

Health-related factors included BMI, smoking status, physical activity, and marital status at or before study baseline in 2008. We did not time-update these factors, as illness preceding death could lead to weight loss, smoking cessation, and reduced physical activity. Self-reported height in 1989 and weight in 2007 were used to calculate BMI, coded with continuous and squared terms, as this produced the best-fitting model. Smoking was assessed biennially through 2007 and coded as never; past smoker of 1 to 4, 5 to 14, 15 to 24, 25 to 34, 35 to 44, or 45 or more cigarettes per day; or present smoker of 1 to 4, 5 to 14, 15 to 24, 25 to 34, 35 to 44, or 45 or more cigarettes per day. In 2005, respondents reported their mean time spent per week in 10 different recreational activities (eg, swimming or walking). Past-year physical activity was calculated in metabolic-equivalent hours per week from these responses. Respondents reported current marital status in 2008 as married, divorced, separated, widowed, in domestic partnership, or single. We considered parental socioeconomic status during the respondent’s infancy, reported in 2005, as a potential confounder. Highest occupation (ie, jobs that usually have higher status and pay) of either parent during the respondent’s infancy was reported as farmer, laborer, blue-collar (eg, mechanic or bus driver) or lower white-collar worker (ie, secretarial or clerical work), or managerial or professional. Parental education was reported as high school or above, some college, or college graduate or above, and parental home ownership in respondent’s infancy was coded as yes or no. In 2005, respondents indicated their race/ethnicity by selecting 1 or more of the following: White, Black or African American, American Indian or Alaska Native, Native Hawaiian or Pacific Islander, or other. For analyses, race was coded White or non-White, as 50 137 individuals in the sample (97.2%) selected only White. Age was measured in months.

### Statistical Analysis

We examined the distribution of health-related factors and covariates by PTSD and depression status in 2008. We then calculated the association of health-related factors with mortality by fitting a single Cox proportional hazard model with all factors included as independent variables, adjusted for age and race/ethnicity. To investigate the association of PTSD and depression with mortality, we first examined the association of PTSD with depression. We then ascertained the best-fitting model, using the Akaike information criterion^48^ to compare 4 models: PTSD alone; depression alone; PTSD and depression; and PTSD, depression, and a PTSD-depression interaction term, using indicator variables as previously described. Finally, we fit 2 Cox proportional hazard models using the best-fitting model, adjusted for age, race/ethnicity, and childhood socioeconomic status and further adjusted for health-related factors, including BMI, smoking status, physical activity, and marital status. In additional analyses, we examined the association of mortality with depression and PTSD symptoms coded continuously, with an interaction term calculated by multiplying the 2 continuous variables, among women exposed to a traumatic event.

To reduce concerns that illness caused both PTSD or depressive symptoms and death, we excluded 3026 women who reported serious illness as their worst trauma and excluded the first year of person-time after the 2008 PTSD questionnaire, meaning we excluded 52 women who died during that year. We excluded an additional 7 women who did not respond to questionnaires between 2009 and 2017, leaving 51 602 women in the sample. For all models, hazard ratios (HRs) were estimated using the phreg procedure in SAS statistical software version 9.4 (SAS Institute). A 2-sided *P* < .05 was considered significant in statistical tests. Data analysis was performed from September 2018 to November 2020.

To improve power, we examined cause of death in 3 aggregated groups: women with no depression or PTSD symptoms, women with any (1-7) PTSD symptoms or with depression but not both, and women with any PTSD symptoms and depression. We tested differences between the reference group of women with no depression or PTSD symptoms and each of the other 2 groups using χ^2^ tests.

## Results

At baseline in 2008, this study included 51 602 women, with mean (range) age 53.3 (43-64) years; 50 137 women (97.2%) were White. The 2093 women with co-occurring high PTSD symptoms and probable depression, compared with 8890 women with no trauma exposure and no depression, were more likely to be divorced or separated (519 women [24.8%] vs 717 women [8.1%]), have obesity (699 women ([33.4%] vs 1985 women [22.3%]), and be current smokers (235 women [11.2%] vs 389 women [4.4%]). Women with high PTSD and probable depression were also more likely, compared with 1926 women with high PTSD symptoms and no depression, to be divorced or separated (295 women [15.3%]), have obesity (508 women [26.4%]), and be current smokers (131 women [6.8%]). Additionally, women with high PTSD symptoms and probable depression, compared with 1700 women with depression and no PTSD symptoms, were more likely to be divorced or separated (250 women [14.7%]), have obesity (521 women [30.6%]), and be current smokers (142 women [8.4%]). The 10 529 women with probable depression, regardless of PTSD symptoms, had higher risk of low physical activity compared with 41 073 women without probable depression (2066 women [19.6%] vs 5729 women [14.0%]) (Table 1). Depression and PTSD were not associated with age at interview or race/ethnicity. In a mutually adjusted model, past and current smoking status vs never-smoked status, higher BMI, less physical activity, and divorced or separated status or single status vs married status were associated with increased mortality. For example, current smoking was associated with more than 2-fold increased risk of death (HR, 2.81; 95% CI, 2.18-3.61), being divorced or separated was associated with 30% increased risk of death (HR, 1.30; 95% CI, 1.02-1.65), and low physical activity (ie, less than 3 metabolic equivalents/wk) was associated with nearly 50% increased risk of death compared with high physical activity (ie, 42 or more metabolic equivalents/wk) (HR, 1.49; 95% CI, 1.09-2.04). Childhood socioeconomic status and participant’s race/ethnicity were not associated with increased mortality.

**Table 1.** Health-Related Characteristics at the Time of PTSD and Depression Assessment, Among 51 602 Women

Characteristic^a^	Women, No. (%)									
	Without depression					With depression				
	No trauma (n = 8890)	PTSD symptoms, No.				No trauma (n = 1215)	PTSD symptoms, No.			
		0 (n = 13 385)	1-3 (n = 12 513)	4-5 (n = 4359)	6-7 (n = 1926)		0 (n = 1700)	1-3 (n = 2949)	4-5 (n = 2572)	6-7 (n = 2093)
Age, mean (SD), y	53.2 (4.7)	53.4 (4.7)	53.3 (4.6)	53.3 (4.6)	53.3 (4.5)	53.2 (4.8)	53.3 (4.7)	53.2 (4.6)	53.5 (4.5)	53.6 (4.4)
Race/ethnicity										
White	8649 (97.3)	12 985 (97.0)	12 143 (97.0)	4257 (97.7)	1876 (97.4)	1181 (97.2)	1658 (97.5)	2861 (97.0)	2498 (97.1)	2029 (96.9)
Black	77 (0.9)	166 (1.2)	150 (1.2)	32 (0.7)	8 (0.4)	9 (0.7)	17 (1.0)	28 (0.9)	28 (1.1)	21 (1.0)
Asian	134 (1.5)	183 (1.4)	150 (1.2)	32 (0.7)	25 (1.3)	23 (1.9)	15 (0.9)	32 (1.1)	18 (0.7)	17 (0.8)
Childhood socioeconomic status										
Parent education, ≤high school	4794 (53.9)	7371 (55.1)	6544 (52.3)	2298 (52.7)	998 (51.8)	710 (58.4)	986 (58.0)	1622 (55.0)	1357 (52.8)	1136 (54.3)
Parent occupation, blue collar	3979 (44.8)	6195 (46.3)	5843 (46.7)	2012 (46.2)	901 (46.8)	579 (47.7)	833 (49.0)	1419 (48.1)	1147 (44.6)	1001 (47.8)
Parent owned home	4743 (53.4)	7066 (52.8)	6306 (50.4)	2203 (50.5)	974 (50.6)	595 (49.0)	824 (48.5)	1472 (49.9)	1279 (49.7)	1022 (48.8)
BMI										
<25	3998 (45.0)	5930 (44.3)	5399 (43.1)	1868 (42.9)	786 (40.8)	489 (40.2)	604 (35.5)	1032 (35.0)	904 (35.1)	732 (35.0)
25 to <30	2543 (28.6)	3808 (28.4)	3565 (28.5)	1224 (28.1)	549 (28.5)	350 (28.8)	495 (29.1)	839 (28.5)	721 (28.0)	570 (27.2)
≥30	1985 (22.3)	3171 (23.7)	3096 (24.7)	1106 (25.4)	508 (26.4)	326 (26.8)	521 (30.6)	967 (32.8)	854 (33.2)	699 (33.4)
Smoking^b^										
Never	6386 (71.8)	9114 (68.1)	8312 (66.4)	2765 (63.4)	1147 (59.6)	814 (67.0)	1060 (62.4)	1807 (61.3)	1520 (59.1)	1152 (55.0)
Former	2115 (23.8)	3563 (26.6)	3551 (28.4)	1323 (30.4)	648 (33.6)	322 (26.5)	498 (29.3)	878 (29.8)	817 (31.8)	706 (33.7)
Current	389 (4.4)	708 (5.3)	650 (5.2)	271 (6.2)	131 (6.8)	79 (6.5)	142 (8.4)	264 (9.0)	235 (9.1)	235 (11.2)
Physical activity, lowest category (<3 metabolic equivalent h/wk)^c^	1249 (14.0)	1939 (14.5)	1711 (13.7)	553 (12.7)	277 (14.4)	234 (19.3)	357 (21.0)	553 (18.8)	511 (19.9)	411 (19.6)
Marital status^d^										
Married	7092 (79.8)	10 746 (80.3)	9935 (79.4)	3164 (72.6)	1361 (70.7)	823 (67.7)	1201 (70.6)	2057 (69.8)	1663 (64.7)	1229 (58.7)
Divorced or separated	717 (8.1)	1216 (9.1)	1258 (10.1)	643 (14.8)	295 (15.3)	202 (16.6)	250 (14.7)	500 (17.0)	527 (20.5)	519 (24.8)
Never married	533 (6.0)	560 (4.2)	576 (4.6)	213 (4.9)	95 (4.9)	97 (8.0)	105 (6.2)	204 (6.9)	175 (6.8)	138 (6.6)
Widowed	139 (1.6)	185 (1.4)	286 (2.3)	180 (4.1)	88 (4.6)	33 (2.7)	40 (2.4)	96 (3.3)	133 (5.2)	122 (5.8)

Abbreviations: BMI, body mass index (calculated as weight in kilograms divided by height in meters squared); PTSD, posttraumatic stress disorder.

^a^ Parents’ education and occupation were assessed in 2005; BMI, smoking, and physical activity were assessed in 2007; marital status was assessed in 2008.

^b^ Data missing for 56 women (0.1%).

^c^ Data missing for 2021 women (3.9%).

^d^ Data missing for 2206 women (4.3%).

We found that PTSD was associated with depression. While probable depression was reported in 1215 of 10 105 women (12%) with no trauma exposure, depression was reported in 2949 of 15 462 women (19%) with 1 to 3 PTSD symptoms, 2572 of 6931 women (37%) with 4 to 5 PTSD symptoms, and 2093 of 4019 women (52%) with 6 to 7 PTSD symptoms. Among 51 609 women, 555 deaths occurred. Women with comorbid PTSD and depression were substantially more likely to die during follow-up compared with women with no trauma exposure and no depression: among 2093 women with high PTSD symptoms and depression, 57 women (2.7%) died, while among 8890 women with no trauma or depression, 63 women (0.7%) died. The best-fitting model of mortality included PTSD, probable depression, and a PTSD-depression interaction term (*P* for interaction = .06). In models adjusted for age, race/ethnicity, and childhood socioeconomic indicators, co-occurring PTSD and depression were associated with mortality. Women with high PTSD symptoms and depression were at nearly 4-fold increased risk of death compared with women with no trauma exposure or depression (HR, 3.80; 95% CI, 2.65-5.45; *P* < .001). Women with depression and moderate PTSD symptoms (HR, 2.03; 95% CI, 1.35-3.03; P < .001) and depression and subclinical PTSD symptoms (HR, 2.85; 95% CI, 1.99-4.07; *P* < .001) were also at increased risk of death compared with women with no trauma or depression (Table 2). With further adjustment for health factors, women with high PTSD symptoms and depression remained at increased risk of death (HR, 3.11; 95% C, 2.16-4.47; *P* < .001) (Table 2). Women with subclinical PTSD symptoms without probable depression had increased risk of death compared with women with no trauma or depression (HR, 1.43; 95% CI, 1.06-1.93; *P* = .02) (Table 2). Depression in women without trauma exposure was associated with more than 2-fold increased risk of death (HR, 2.39; 95% CI, 1.44-3.95; *P* < .001). However, risk of mortality among women with depression and trauma exposure who did not develop PTSD symptoms was not increased compared with the reference group (HR, 1.28; 95% CI, 0.74-2.21; *P* = .39) (Table 2).

**Table 2.** Association of Trauma Exposure and PTSD Symptoms With Mortality, by Depression Status, Adjusted for Health Factors^a^

Trauma and PTSD	Deaths/person-years	Hazard ratio (95% CI)				
		Model 1^b^	Model 2^c^	Model 3^d^	Model 4^e^	Model 5^f^
Without depression						
No trauma	63/76 872	1 [Reference]	1 [Reference]	1 [Reference]	1 [Reference]	1 [Reference]
Trauma, no PTSD	112/115 673	1.15 (0.84-1.56)	1.14 (0.84-1.55)	1.15 (0.84-1.57)	1.13 (0.83-1.54)	1.15 (0.84-1.56)
PTSD symptoms, No.						
1-3	130/108 220	1.43 (1.06-1.93)^g^	1.38 (1.02-1.87)^g^	1.41 (1.05-1.91)^g^	1.39 (1.03-1.89)^g^	1.44 (1.07-1.95)^g^
4-5	44/37 615	1.39 (0.95-2.05)	1.30 (0.88-1.91)	1.37 (0.93-2.01)	1.33 (0.91-1.96)	1.41 (0.96-2.07)
6-7	17/16 603	1.27 (0.75-2.18)	1.13 (0.66-1.95)	1.23 (0.72-2.11)	1.19 (0.69-2.03)	1.28 (0.75-2.20)
With depression						
No trauma	20/10 441	2.39 (1.44-3.95)^h^	2.23 (1.34-3.70)^i^	2.36 (1.43-3.92)^h^	2.27 (1.37-3.76)^i^	2.32 (1.40-3.85)^i^
Trauma, no PTSD	16/14 642	1.28 (0.74-2.21)	1.14 (0.66-1.98)	1.23 (0.71-2.15)	1.19 (0.68-2.06)	1.23 (0.71-2.13)
PTSD symptoms, No.						
1-3	58/25 336	2.85 (1.99-4.07)^h^	2.52 (1.76-3.61)^h^	2.77 (1.94-3.98)^h^	2.62 (1.83-3.75)^h^	2.76 (1.93-3.94)^h^
4-5	38/22 120	2.03 (1.35-3.03)^h^	1.63 (1.08-2.46)^g^	1.87 (1.24-2.80)^i^	1.84 (1.22-2.75)^i^	1.96 (1.30-2.93)^i^
6-7	57/17 827	3.80 (2.65-5.45)^h^	3.11 (2.16-4.47)^h^	3.57 (2.49-5.13)^h^	3.40 (2.67-4.89)^h^	3.65 (2.54-5.24)^h^

Abbreviations: BMI, body mass index; PTSD, posttraumatic stress disorder.

^a^ All models adjusted for individual’s age in months; individual’s race/ethnicity; and parents’ education, occupation, and home ownership in individual’s infancy. Smoking was coded as never; past 1-4, 5-14, 15-24, 25-34, 35-44, or 45 or more cigarettes/d; current 1-4, 5-14, 15-24, 25-34, 35-44, or 45 or more cigarettes/d; or missing smoking status. Physical activity was coded as less than 3, 3 to less than 9, 9 to less than 18, 18 to less than 27, 27 to less than 42, or 42 or more metabolic equivalents/wk or missing exercise data. Marital status was coded as married, divorced or separated, never married, widowed, or missing marital status. BMI was coded as a continuous variable. BMI and BMI-squared terms were included.

^b^ Adjusted for childhood socioeconomic factors, race, and age.

^c^ Model 1 further adjusted for BMI, smoking, physical activity, and marital status.

^d^ Model 1 further adjusted for BMI only.

^e^ Model 1 further adjusted for smoking only.

^f^ Model 1 further adjusted for physical activity only.

^g^ *P* < .05.

^h^ *P* < .001.

^i^ *P* < .01.

To further explore which potentially modifiable health factors might account for increased risk of death among women with PTSD and depression, we fit 3 additional models separately adjusted for BMI, smoking, and physical activity. The association of PTSD and depression with mortality remained significant in models adjusted for BMI or smoking. For example, among women with depression and high PTSD symptoms, the HR adjusted for BMI was 3.57 (95% CI, 2.49-5.13; *P* < .001) and the HR adjusted for smoking was 3.40 (95% CI, 2.67-4.89; *P* < .001) (Table 2).

In analyses among women who had trauma exposure, with PTSD and depression symptoms coded continuously, the best-fitting model included PTSD, depression, and an interaction term. In women without depression symptoms, increased number of PTSD symptoms was not associated with increased mortality (HR per PTSD symptom, 0.97; 95% CI, 0.91-1.04; *P* = .42). Depression symptoms and depression-PTSD interaction were associated with increased risk of mortality (HR per depression symptom, 1.03; 95% CI, 1.01-1.06; *P* = .02; HR for interaction, 1.01; 95% CI, 1.00-1.01; *P* = .02).

Among 7565 women with PTSD symptoms and probable depression, 109 deaths (1.4%) occurred for which we obtained cause of death information, compared with 124 such deaths (0.6%) among 22 215 women with no depression or PTSD. Women with PTSD symptoms and probable depression, compared with women with no PTSD or depression, had higher rates of death from cardiovascular disease (17 women [0.22%] vs 11 women [0.05%]), diabetes (4 women [0.05%] vs 0 women), unintentional injury (7 women [0.09%] vs 7 women [0.03%]), suicide (9 women [0.12%] vs 1 woman [<0.01%]), and other causes of death (14 women [0.19%] vs 17 women [0.08%]). The rate of such deaths among women with PTSD and depression did not differ significantly compared with women with PTSD symptoms alone or probable depression alone (Table 3). Deaths from cancer were not significantly different in women with PTSD and depression compared with women with no depression or PTSD symptoms

**Table 3.** Incidence of Causes of Death Over Follow-up by PTSD and Depression, Excluding Deaths Without Cause of Death Information Among 51 406 Women

Cause of death^a^	Women, No. (%)		
	No PTSD symptoms or probable depression (n = 22 215)	PTSD symptoms alone or probable depression alone (n = 21 626)	PTSD symptoms and probable depression (n = 7565)^b^
Cancer	72 (0.32)	89 (0.41)	36 (0.48)
Cardiovascular disease	11 (0.05)	16 (0.07)	17 (0.22)^c^
Respiratory disease	7 (0.03)	2 (0.01)	6 (0.08)
Diabetes	0 (0.00)	1 (0.00)	4 (0.05)^c^
Unintentional injury	7 (0.03)	8 (0.04)	7 (0.09)^d^
Suicide	1 (0.00)	4 (0.02)	9 (0.12)^c^
Other causes of death	17 (0.08)	21 (0.10)	14 (0.19)^d^
All deaths with cause of death information	124 (0.56)	151 (0.69)	109 (1.44)^c^

Abbreviation: PTSD, posttraumatic stress disorder.

^a^ Percentages shown are small, because 384 individuals (0.7%) with cause of death information died during follow-up.

^b^ *P* values calculated from χ^2^ compared with no PTSD symptoms or probable depression group.

^c^ *P* < .001.

^d^ *P* < .05.

## Discussion

This cohort study, to our knowledge the first large study of co-occurring PTSD and depression, found that women with co-occurring high PTSD symptoms and probable depression had nearly 4-fold increased mortality risk compared with women with no trauma exposure and no depression. These findings are particularly salient given that women have 2-fold the lifetime prevalence of PTSD and depression compared with men.^24,25,26^ A portion of the association in this study was accounted for by health risk factors, in particular, smoking and BMI, although the association remained even after accounting for major health risk factors. Women with PTSD symptoms and depression had increased incidence of death across nearly all major causes of death. These findings mirror a 2006 study^16^ of military veterans showing broadly increased risk of death in individuals with PTSD, including from cardiovascular illness, cancer, unintentional and intentional injuries, as well as a large Danish population study^49^ indicating that suicide alone does not account for the increased risk of death in individuals with depression.

Among women with depression in our study, those who were exposed to a traumatic event but did not develop PTSD symptoms were not at increased risk of death compared with women with no trauma exposure or depression, while other women with depression, including those with no exposure to trauma, were at increased risk. It may be that not developing PTSD symptoms after experiencing trauma is an indicator associated with psychological resilience^50^ and this resilience may be protective against the physical health effects of depression.

Symptoms of PTSD and depression overlap, with dysphoria and numbing common to both disorders.^51^ Questions have long been raised as to whether specific psychiatric diagnoses map to distinct phenomena or whether instead there are domains of dysfunction that span disorders (eg, Research Domain Criteria^52^), or even a single underlying psychopathology factor.^53,54,55^ Regardless, when PTSD and depression co-occur, it likely indicates more severe distress.^37,51,56^ Our findings are consistent with prior studies that found that co-occurring PTSD and depression are associated with worse health outcomes compared with either disorder alone. In military veterans and the general population, PTSD with depression has been associated with greater risk of suicidal behaviors^57^ and suicidal ideation^58^ compared with PTSD or depression alone. A 2004 study^59^ of Bosnian refugees found that those with comorbid PTSD and depression had greater social impairment, global dysfunction, and occupational disability compared with refugees with PTSD alone. In studies of US war veterans,^60,61^ comorbid PTSD and depression have been associated with reduced quality of life and impaired life satisfaction, compared with either disorder alone. In addition, there is evidence that the biological stress response is distinct among individuals with PTSD and depression compared with individuals with PTSD alone,^62,63,64,65^ which may contribute to these worse health outcomes.

### Limitations

Our study has several limitations. Our sample included predominantly White women ages 43 to 64 years, which may limit generalizability. The sample also included only respondents who survived until the PTSD questionnaire was administered, which may have attenuated associations.^66^ In addition, our measures captured symptoms of both disorders rather than clinical diagnoses, lifetime PTSD symptoms were queried retrospectively, and only past-week depressive symptoms were queried, which may have resulted in misclassification. We lacked information on illicit substance use and abuse, which have been associated with increased risk of death in veterans with PTSD.^19,20^ Illicit substance use may have accounted for an additional part of the association of PTSD and depression with mortality beyond the health factors we examined.

## Conclusions

The findings of this cohort study suggest that treatment of PTSD and depression in women with symptoms of both disorders and efforts to improve their health behaviors may reduce this population’s increased risk of mortality. Our results suggest that future investigations of the associations among PTSD, depression, and physical health outcomes should consider risk associated with co-occurrence of the disorders rather than modeling risk associated with 1 disorder adjusted for the other. Our findings additionally highlight the need for better access to and dissemination of effective treatments for comorbid PTSD and depression.^67,68^
